# Propolis as a Natural Remedy in Reducing Dental Plaque and Gingival Inflammation: A Systematic Review and Meta-Analysis

**DOI:** 10.3390/jfb16090336

**Published:** 2025-09-08

**Authors:** Magdalena Sycińska-Dziarnowska, Liliana Szyszka-Sommerfeld, Monika Bugajska, Magdalena Ziąbka, Izabela Szućko-Kociuba, Gianrico Spagnuolo, Krzysztof Woźniak, Hyo-Sang Park

**Affiliations:** 1 Department of Maxillofacial Orthopaedics and Orthodontics, Pomeranian Medical University in Szczecin, Al. Powst. Wlkp. 72, 70111 Szczecin, Poland; 2 Laboratory for Propaedeutics of Orthodontics and Facial Congenital Defects, Pomeranian Medical University in Szczecin, Al. Powst. Wlkp. 72, 70111 Szczecin, Poland; 3 Private Dental Practice, Witolda Pileckiego 12, 07410 Ostrołęka, Poland; 4 Department of Ceramics and Refractories, Faculty of Materials Science and Ceramics, AGH University of Krakow, al. A. Mickiewicza 30, 30059 Krakow, Poland; ziabka@agh.edu.pl; 5 Institute of Biology, University of Szczecin, Wąska 13, 71415 Szczecin, Poland; 6 Department of Neurosciences, Reproductive and Odontostomatological Sciences, University of Naples “Federico II”, 80131 Napoli, Italy; gspagnuo@unina.it; 7 School of Dentistry, College of Dental Medicine, Kaohsiung Medical University, Kaohsiung 80708, Taiwan; 8 Department of Orthodontics, College of Dentistry, Kyungpook National University, Daegu 41940, Republic of Korea; parkhs@knu.ac.kr

**Keywords:** propolis, gingival inflammation, plaque index, mouthwash, toothpaste

## Abstract

Dental plaque, if not regularly removed through proper oral hygiene, can lead to tooth decay, gingivitis, and more severe periodontal disease. Effective plaque removal is essential in preventing gingivitis, the precursor to periodontitis. Propolis, a bee product known for its antibacterial, anti-inflammatory, and antioxidant properties, has shown potential in dental applications. This systematic review and meta-analysis was conducted to evaluate the efficacy of propolis-containing mouthwashes and toothpastes in reducing dental plaque and gingival inflammation. Materials and Methods: The study protocol was registered in PROSPERO (CRD42023467573), and the review was conducted in accordance with PRISMA guidelines. A comprehensive search of PubMed, PubMed Central, Embase, Scopus, and Web of Science was performed up to 10 May 2025 to identify randomized controlled trials and observational studies assessing propolis-based mouthwashes or toothpastes. Data synthesis used random-effects meta-analysis due to anticipated heterogeneity among studies. Results: Seven randomized controlled trials were included in the meta-analysis, evaluating the efficacy of propolis alcohol-free mouthwash on plaque index (PI) and gingival index (GI). For PI, the pooled standardized mean difference (SMD) was 1.74 (95% CI: 0.19–3.29; *p* = 0.036), with low between-study heterogeneity (I^2^ = 13.7%). For GI, the pooled SMD was 2.19 (95% CI: 1.10–3.29; *p* = 0.005), with no observed heterogeneity (I^2^ = 0.0%). Propolis mouthwashes demonstrated large effect sizes, significantly reducing plaque accumulation and gingival inflammation compared to baseline. Conclusions: The evidence supports the potential of propolis-containing mouthwashes and toothpastes in managing dental plaque and gingival health. Propolis-based oral care products could be a valuable addition to preventive strategies in dental hygiene, offering an alternative for reducing dental plaque and gingival inflammation.

## 1. Introduction

Dental plaque is a type of biofilm composed of diverse microorganisms, making it a heterogeneous structure [1,2]. As long as proper saliva flow is maintained and oral hygiene is upheld, biofilm serves as a protective barrier against external pathogens. However, when these conditions are disrupted, developing plaque interferes with normal saliva flow, promoting colonization by pathogenic microorganisms. If the bacteria in dental plaque are exposed to continuous or intermittent sugar intake, they produce acid, leading to a decrease in pH and resulting in enamel demineralization [1,3].

Inadequate removal of bacterial plaque significantly contributes to the development of gingivitis, an inflammation of the tissue surrounding the teeth. This is considered the first step for the later development of periodontitis and the progressive loss of attachment around the teeth. Therefore, control of gingival inflammation is a fundamental strategy for preventing periodontitis and preventing its recurrence [4,5]. Efforts to control and combat caries can focus on inhibiting biofilm development (e.g., preventing the adhesion of cariogenic bacteria, manipulating cell signaling mechanisms, delivering effective antibacterial substances) or enhancing host defenses [1].

A well-known antibacterial substance is propolis, a bee product with a wide spectrum of biological properties. The word propolis comes from Greek, meaning “before the city,” referring to the use of this substance by bees to strengthen the outer walls of the hive by sealing gaps, smoothing walls, and maintaining constant humidity and temperature inside the hive throughout the year. This allows for maintaining hive homeostasis by limiting microbial growth and regulating airflow. Bees also use propolis to defend against intruders. It has antibacterial properties that help combat bee pathogens such as *Paenibacillus larvae*, *Ascosphaera apis*, and *Nosema ceranae* [6,7,8,9].

Bee glue is produced from tree buds collected by bees and then digested. The substance is secreted by bee glands, and its chemical composition depends on the type of plant. Propolis is often dark in color, most commonly brown or green [9]. Raw propolis typically consists of 50% plant resins, 30% waxes, 10% essential and aromatic oils, 5% pollen, and 5% other organic substances [10]. To date, over 500 chemical compounds have been isolated from propolis, mainly belonging to the classes of flavonoids, terpenoids, and phenolic acids [11]. However, the content of vitamins, minerals, and other components can vary depending on the type of plant from which the pollen originates and the location of the propolis [12]. Furthermore, researchers have shown that the chemical composition and quality of propolis significantly depend on the species of bee that produced it [13].

Propolis is used in medicine and dentistry due to its chemical composition and medicinal properties [14]. The phenolic compounds and flavonoids in propolis exhibit strong antioxidant properties [15]. Propolis also exhibits anti-inflammatory, anticancer, and antiproliferative antibacterial and antiviral properties [16,17,18].

Given this wide range of effects, the potential of propolis in dentistry, oral health prevention, and medicine cannot be overstated. The effectiveness of propolis in reducing dental plaque and combating gingivitis was verified in several clinical studies [19]. The propolis extract used as mouthwash or in gels and capsules reveals anti-plaque activity and improves gingival health [20]. Sparabombe et al. evaluated the anti-inflammatory effect of propolis-based mouthwashes during a three-month treatment and showed a significant improvement in the reduction of plaque buildup and gingival bleeding [21]. Giammarinaro et al. evaluated the effectiveness of a propolis and herbal formula, compared with chlorhexidine-based formulas [22]. They proved that patients treated with propolis had better results in reducing oxidative stress. In contrast, Tanasiewicz et al. assigned an influence of the application of toothpaste with propolis extract in ethanol on the state of the oral cavity, and after eight weeks of treatment no significant difference in comparison to the first week was observed [23].

Despite the availability of chemical agents such as chlorhexidine for plaque control, their long-term use is often associated with undesirable side effects, including tooth staining, taste disturbances, and mucosal irritation. These limitations underscore the need for safer, natural alternatives that can effectively manage plaque and gingival inflammation. Propolis, with its diverse bioactive compounds, exhibits antibacterial, anti-inflammatory, and antioxidant properties, making it a promising candidate for oral health care. However, there remains a lack of high-quality evidence consolidating its efficacy across different populations, formulations, and clinical settings. This systematic review and meta-analysis aims to address these gaps by evaluating the effectiveness of propolis-based mouthwashes and toothpastes in reducing dental plaque and gingival inflammation.

However, the significant variation in the composition of this substance, which depends on the local flora, harvest time, environmental pollution levels, presence of contaminating waxes, and even unpredictable factors such as weather conditions, makes studying the therapeutic applications of propolis challenging. This variability makes it difficult to define a uniform standard for medical use. The use of different extraction methods and solvents in various preclinical studies is also significant. With the overarching goal of developing effective measures to control gingivitis, this systematic review and meta-analysis investigates the following research question: What is the current evidence on the effectiveness of propolis mouthwashes and toothpastes in reducing dental plaque and gingival inflammation?

## 2. Materials and Methods

**Figure 1:**
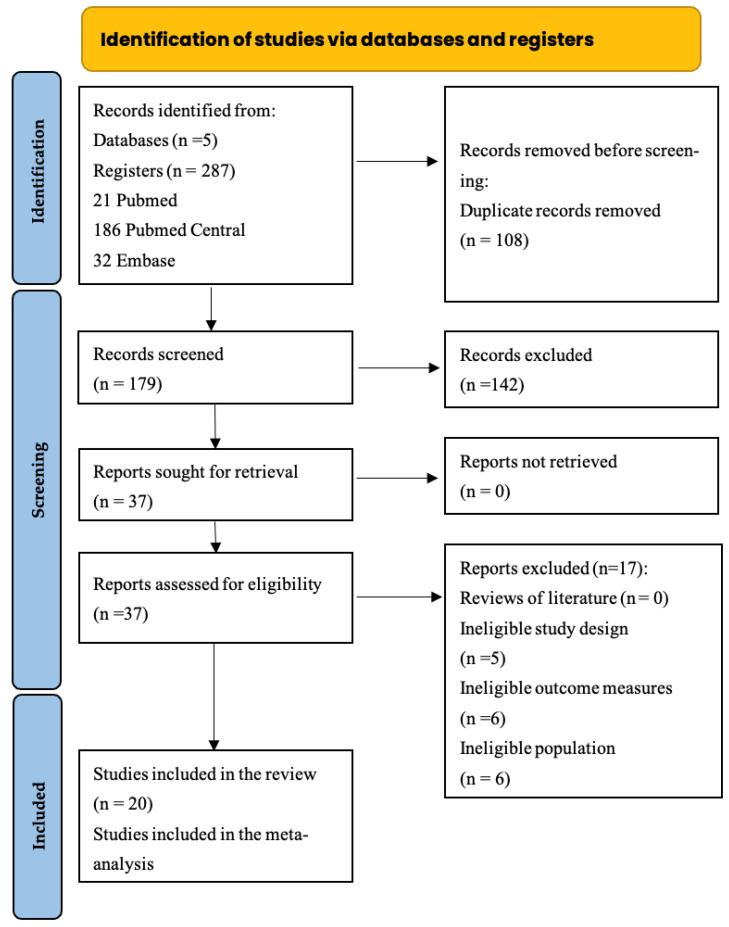
Systematic review flow chart according to PRISMA Statement.

Firstly, to enhance compliance with systematic review guidelines, the study protocol was registered in PROSPERO (CRD42023467573). The screening process was visually represented through the PRISMA diagram (Figure 1) [24].

Pubmed (“propolis”) AND (“gingival index” OR “plaque index” OR “Gingival Index” OR “Plaque Index”) AND (“mouthwash*” OR “mouthrinse” OR “toothpaste”)

Pubmed Central (“propolis” [All Fields]) AND (“gingival index” [All Fields] OR “plaque index” [All Fields]) AND (“mouthwash*” [All Fields] OR “mouthrinse” [All Fields] OR “toothpaste” [All Fields])

Embase (‘propolis’/exp OR propolis) AND (‘gingival index’ OR ‘plaque index’) AND (mouthwash* OR mouthrinse OR toothpaste)

Scopus TITLE-ABS-KEY (propolis) AND (“gingival index” OR “plaque index”) AND (mouthwash* OR mouthrinse OR toothpaste)

Web of Science TS = (propolis) AND (“gingival index” OR “plaque index”) AND (mouthwash* OR mouthrinse OR toothpaste)

### 2.1. Search Strategy

Two reviewers systematically and independently conducted a thorough search of multiple databases, namely PubMed, PubMed Central, Embase, Scopus, and Web of Science, with no restrictions on publication date. The search inquiry (“propolis”) AND (“gingival index” OR “plaque index” OR “Gingival Index” OR “Plaque Index”) AND (“mouthwash*” OR “mouthrinse” OR “toothpaste”), initially prepared for PubMed, was subsequently adapted for application to other databases. After a detailed search, all duplicate records were removed.

The current systematic review was structured according to the PICO framework [25], focusing on the following. Population: People who were in good general health, aged 15 years or older, with no periodontal disease. Not using removable dentures. Intervention: Mouthwashes or toothpastes containing propolis, regardless of concentration. Comparison: Standard oral care products or placebo. Outcomes: The primary outcomes were the measurement of gingival inflammation and/or plaque index. The research question addressed was as follows: How do propolis mouthwashes or toothpastes reduce gingival inflammation, based on the gingival index (GI) and plaque index (PI)?

The literature search was completed on 10 May 2025, with no limitations on publication dates to ensure a comprehensive review of relevant articles. The review process was carried out in an unbiased manner to ensure objectivity.

### 2.2. Eligibility Criteria

Inclusion Criteria: Randomized controlled trials (RCTs) and controlled clinical trials (CCTs), and observational studies (including case-control and cohort studies) involving the use of propolis mouthwashes/mouth rinses or toothpastes.

Exclusion Criteria: Literature reviews, systematic reviews, case reports, animal studies, unpublished data, and in vitro studies. Research with juveniles under the age of 15, patients with additional diseases or craniofacial abnormalities, or studies using non-propolis-related interventions.

### 2.3. Data Extraction

Firstly, duplicate records were excluded. The first reviewer (M.S.-D.) systematically assessed the abstracts and titles of the studies. Subsequently, the second reviewer (L.S.-S.) re-examined all records to confirm potentially eligible studies. Full-text articles that passed the initial screening were then thoroughly reviewed, and decisions about inclusion or exclusion were determined according to predefined criteria. Whenever uncertainties or ambiguities arose, they were resolved through discussion among the two reviewers and a third author (M.B.), ensuring a comprehensive and unbiased review. For consistency and transparency, a spreadsheet was created following the Cochrane Collaboration guidelines to facilitate comparative analysis of the selected studies. Agreement among reviewers was measured using Cohen’s Kappa coefficient.

### 2.4. Quality Assessment

For randomized controlled trials, version 2 of the Cochrane Risk of Bias tool (RoB2) was applied, while cross-over trials were evaluated using the specialized Cochrane Risk of Bias Tool for Cross-Over Trials [26]. Non-randomized studies were appraised through the Newcastle–Ottawa Scale (NOS) [27]. The evaluation was performed independently by two reviewers (M.S.-D. and L.S.-S.). Any uncertainties or disagreements encountered during the process were discussed with a third author (M.B.). Inter-rater reliability was quantified using Cohen’s Kappa statistic.

### 2.5. Statistical Analysis

The level of statistical significance was predefined as α = 0.05 for all hypothesis tests. The primary effect size metric was the standardized mean difference (SMD), calculated as Hedges’ g to account for small-sample bias correction, which quantifies the magnitude of treatment effects on plaque and gingival indices. Descriptive statistics, including means (M) and standard deviations (SD), were extracted from individual studies to characterize baseline and post-treatment values, facilitating computation of within-study variances.

#### 2.5.1. Identification of Influential Studies

To assess the robustness of the meta-analytic results and detect potential outliers or influential studies, influence diagnostics were performed. This approach computes leave-one-out diagnostics for each study, including (1) the externally standardized residual, which measures the deviation of a study’s effect size from the pooled estimate after exclusion; (2) the DFFITS value, indicating the change in the fitted pooled effect upon omission; (3) Cook’s distance, quantifying the overall influence on the summary estimate; (4) the covariance ratio, evaluating the impact on parameter precision; (5) the leave-one-out amount of residual heterogeneity (τ^2^), reflecting changes in between-study variance; (6) the leave-one-out test statistic for residual heterogeneity (Q statistic), assessing shifts in heterogeneity significance; and (7) DFBETAS values, estimating the influence on individual model coefficients. Thresholds for identifying influential cases included studentized residuals exceeding ± 2.0, Cook’s distance > 1.0, and DFFITS values suggesting substantial shifts. Additionally, the diagonal elements of the hat matrix were examined alongside leave-one-out estimates of the I^2^ statistic, which quantifies the proportion of total variability attributable to between-study heterogeneity.

#### 2.5.2. Pooled Effect Estimation and Between-Study Heterogeneity

Pooled effect sizes were estimated using a random-effects model to account for anticipated between-study heterogeneity. The inverse-variance method was employed for weighting studies, where each study’s contribution is proportional to the inverse of its variance, incorporating both within- and between-study components. Between-study variance (τ^2^) was estimated via the Paule–Mandel method, selected for its empirical performance in balancing bias and precision. Confidence intervals for τ^2^ and its square root (τ) were derived using the Q-profile method, an iterative profiling technique that leverages the distribution of the Q statistic. To adjust for uncertainty in τ^2^ estimation and improve coverage probabilities, the Hartung–Knapp method was applied, utilizing a t-distribution for inference on the pooled SMD. Heterogeneity was further quantified with the I^2^ statistic (expressed as a percentage) and the H statistic (ratio of total to within-study variability), alongside Cochran’s Q test for statistical significance. Results were visualized in forest plots, displaying individual study SMDs, 95% confidence intervals, weights, and the overall pooled estimate with its prediction interval.

#### 2.5.3. Assessment of Publication Bias

Publication bias was evaluated using the linear regression test for funnel plot asymmetry (Thompson’s test), based on a weighted linear regression of the treatment effect on its standard error using an additive between-study variance component denoted as methods. The test incorporated an additive residual heterogeneity variance assumption (τ^2^ = 0 for the bias model), with the Paule–Mandel estimator applied to any residual τ^2^ in the underlying model. The predictor variable was the standard error of the effect size, and weights were assigned via the inverse-variance method to prioritize more precise studies. A *p*-value < 0.05 from the test on the intercept indicated significant asymmetry, suggestive of small-study effects or publication bias. Funnel plots were generated to visually inspect symmetry, plotting effect sizes against standard errors, with asymmetry potentially reflecting selective reporting.

#### 2.5.4. Statistical Environment

Analyses were conducted using the R Statistical language (version 4.3.1; R Core Team, 2023) on Windows 10 Pro 64 bit (build 19045). 

## 3. Results

### 3.1. Search Strategy and Study Selection

The literature search identified 287 articles across multiple databases: 21 from PubMed, 186 from PubMed Central, 32 from Embase, 26 from Scopus, and 22 from Web of Science. Following the removal of 108 duplicates, the remaining records were screened in detail. Ultimately, 20 studies were included in the qualitative synthesis.

The selection process was illustrated using a PRISMA flow diagram (Figure 1). Agreement between reviewers demonstrated strong consistency, with a Cohen’s Kappa coefficient of 0.96, indicating a very high level of inter-rater reliability. This degree of concordance reinforces the overall validity and robustness of the systematic review findings.

### 3.2. Mouthwash Results

As presented in Table 1, propolis mouthwash was shown to be effective in reducing PI, GI often performing comparably to CHX. In studies where propolis mouthwash was compared to CHX, both treatments resulted in significant improvements in PI and GI, with some studies indicating propolis’s superior effectiveness in reducing gingival inflammation. Propolis mouthwash was also significantly better than placebo in decreasing plaque and gingival bleeding.

### 3.3. Toothpaste Results

As stated in Table 1, toothpaste containing propolis demonstrated significant efficacy in reducing plaque and gingival inflammation compared to regular or control toothpaste. Studies indicate that propolis toothpaste results in lower Modified Gingival and Plaque Index (MGMPI) scores and greater reductions in plaque index PI compared to conventional toothpaste brands. Propolis toothpaste also showed superior results in reducing gingival bleeding indices, highlighting its potential for improving overall periodontal health.

### 3.4. Quality Assessment

Cohen’s Kappa coefficient was calculated at 0.95, indicating a high level of agreement between the authors. The quality assessment of the RCTs was shown in Table 2.

The cross-over design studies by Bhat et al. [41] and Ranjan et al. 2023 [45] were assessed using the Cochrane Risk of Bias Tool for Cross-Over Trials presenting overall low risk of bias. Sequence Generation: low risk of bias, Allocation Concealment: low risk of bias, Blinding of Participants and Personnel: low risk of bias, Blinding of Outcome Assessors: low risk of bias in Bhat et al. study [41] and some concerns in Ranjan et al. 2023 [45], Carry-Over Effects: low risk of bias, Period Effects: some concerns, Washout Period: low risk of bias, Handling of Missing Data: some concerns in Bhat et al. study [41] and low risk in Ranjan et al. 2023 [45].

The NOS score for the study by Pereira et al. [38] was evaluated as 8 per 9. The individual categories received the following ratings: 3 for selection, 2 for comparability, and 3 for outcome.

**Table 2:** Evaluation of the quality of the RCTs.

N	Author, Year	Randomization Process	Deviations from the Intended Interventions	Missing Outcome Data	Measurement of the Outcome	Selection of the Reported Results	Overall
1	Amano et al., 2025 [40]	Low risk	Low risk	Low risk	Low risk	Low risk	Low risk
2	Bapat et al., 2021 [28]	Low risk	Low risk	Some concerns	Low risk	Low risk	Low risk
3	Biria et al., 2019 [42]	Some concerns	Some concerns	Low risk	Some concerns	Low risk	Some concerns
4	Dehghani et al., 2019 [29]	Low risk	Low risk	Some concerns	Low risk	Low risk	Low risk
5	Dodwad et al., 2011 [20]	Low risk	Some concerns	Low risk	Some concerns	Some concerns	Some concerns
6	Ercan et al., 2015 [30]	Low risk	Some concerns	Low risk	Low risk	Some concerns	Low risk
7	Fereidooni et al., 2014 [43]	Some concerns	Low risk	Low risk	Some concerns	Low risk	Some concerns
8	Gunjal et al., 2024 [31]	Low risk	Low risk	Low risk	Low risk	Low risk	Low risk
9	Khabazian et al., 2025 [32]	Low risk	Low risk	Low risk	Some concerns	Some concerns	Some concerns
10	Kiani et al., 2022 [33]	Low risk	Low risk	Low risk	Low risk	Some concerns	Low risk
11	Koo et al., 2002 [34]	Low risk	Low risk	Low risk	Some concerns	Low risk	Low risk
12	Kripal et al., 2019 [35]	Low risk	Some concerns	Low risk	High risk	Some concerns	Some concerns
13	Mallikarjun et al., 2022 [36]	Some concerns	High risk	Low risk	High risk	Low risk	High risk
14	Murray et al., 1997 [37]	Some concerns	Some concerns	Low risk	High risk	Low risk	Some concerns
15	Penmetsa et al., 2023 [44]	Some concerns	Low risk	Some concerns	High risk	Some concerns	Some concerns
16	Porwal et al., 2018 [39]	Some concerns	High risk	Low risk	High risk	Low risk	High risk
17	Suriamah et al., 2019 [46]	Some concerns	Some concerns	Low risk	High risk	Low risk	Some concerns

### 3.5. Characteristics of the Studies Included in the Meta-Analysis

The meta-analysis incorporated data from seven unique randomized controlled trials that fulfilled the predefined quality criteria evaluating the efficacy of propolis alcohol-free mouthwash, published between 2002 and 2025. These studies collectively involved 136–140 participants across both outcomes, with individual sample sizes ranging from 6 to 45. All trials employed a pre–post design over a treatment period, focusing on changes in plaque index and gingival index as primary indicators of oral biofilm control and periodontal inflammation, respectively. Other eligible studies (CCTs and observational studies) were included in the qualitative review but were not pooled quantitatively.

For the plaque index, six studies—Gunjal et al. [31]; Bapat et al. [28]; Dehghani et al. [29]; Kripal et al. [35]; Pereira et al. [38]; and Koo et al. [34]—provided baseline and post-treatment means, encompassing 136 participants. Baseline means ranged from 0.99 (SD 0.12) in Bapat et al. [28] to 2.39 (SD 0.69) in Pereira et al. [38], reflecting diverse starting levels of plaque accumulation, from mild to moderate, which clinically correspond to varying risks of caries development and gingival irritation. Post-treatment means decreased in all studies, ranging from 0.46 (SD 0.09) in Bapat et al. [28] to 1.82 (SD 0.62) in Pereira et al. [38], with absolute reductions between 0.35 and 0.67 units, as shown in Table 3. This consistent decline indicates the mouthwash’s antimicrobial properties, likely attributable to propolis flavonoids, which effectively disrupt oral biofilms, potentially reducing plaque-related pathologies in clinical practice.

For the gingival index, six studies—Pereira et al. [38]; Khabazian et al. [32]; Gunjal et al. [31]; Bapat et al. [28]; Dehghani et al. [29]; and Kripal et al. [35]—reported data on 141 participants. Baseline means varied from 1.03 (SD 0.13) in Bapat et al. [28] to 2.25 (SD 0.37) in Khabazian et al. [32], revealing initial gingival inflammation levels from minimal to moderate, which are associated with increased bleeding on probing and early periodontitis risk. Post-treatment means uniformly decreased, ranging from 0.52 (SD 0.16) in Bapat et al. [28] to 2.00 (SD 0.19) in Khabazian et al. [32], with reductions of 0.25 to 0.66 units, as shown in Table 3.

**Table 3:** Characteristics of studies evaluating the efficacy of propolis alcohol-free mouthwash.

Plaque Index Studies							
Study ID	Citation	Pre-Treatment			Post-Treatment		
		Mean	SD	N	Mean	SD	N
1	Gunjal, 2024 [31]	1.37	0.27	45	0.70	0.25	45
2	Bapat, 2021 [28]	0.99	0.12	30	0.46	0.09	30
3	Dehghani, 2019 [29]	1.40	0.60	18	0.70	0.50	18
4	Kripal, 2019 [35]	1.95	0.07	15	1.47	0.21	15
5	Pereira, 2011 [38]	2.39	0.69	22	1.82	0.62	21
6	Koo, 2002 [34]	1.13	0.14	6	0.78	0.17	6
**Gingival Index Studies**							
**Study ID**	**Citation**	**Pre-Treatment**			**Post-Treatment**		
		**Mean**	**SD**	**N**	**Mean**	**SD**	**N**
1	Pereira, 2011 [38]	1.17	0.20	22	0.70	0.18	21
2	Khabazian, 2025 [32]	2.25	0.37	10	2.00	0.19	10
3	Gunjal, 2024 [31]	1.31	0.24	45	0.65	0.26	45
4	Bapat, 2021 [28]	1.03	0.13	30	0.52	0.16	30
5	Dehghani, 2019 [29]	1.70	0.30	18	1.30	0.60	18
6	Kripal, 2019 [35]	1.86	0.19	15	1.42	0.20	15

### 3.6. Plaque Index Meta-Analysis

#### 3.6.1. Influence Diagnostics Analysis

The meta-analysis of six studies revealed substantial heterogeneity in plaque index outcomes (I^2^ = 60.3% [95% CI: 2.6–83.8%]; H = 1.59 [95% CI: 1.01–2.48]; Q (5) = 12.59, *p* = 0.028), attributable to methodological variations. Influence diagnostics (Figure S1 in Supplementary Materials) identified the Kripal et al. [35] study as exerting undue influence, with a studentized residual of 2.61 (exceeding the threshold of 2.0), Cook’s distance of 1.08 (>1.0), DFFITS of 1.43, and covariance ratio of 0.51 (<1.0). These metrics indicate outlier status and potential distortion of the pooled estimate. Leave-one-out sensitivity analysis (Figure S2 in Supplementary Materials) demonstrated that excluding Kripal et al. [35] reduced heterogeneity to I^2^ = 14%, enhancing result consistency.

#### 3.6.2. Pooled Effect and Between-Study Heterogeneity

**Figure 2:**
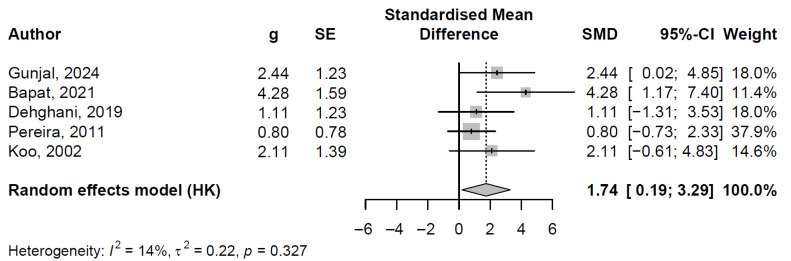
Forest plot of standardized mean differences in plaque index reduction across studies (random effects model) [28,29,31,34,35].

The random-effects model consisted of five randomized controlled trials, encompassing 121 participants, employing Hedges’ g for standardized mean difference (SMD) estimation with Paule–Mandel tau^2^ and Hartung–Knapp adjustment, and yielded a pooled SMD of 1.74 (95% CI: 0.19 to 3.29; t = 3.11, *p* = 0.036), indicating a statistically significant reduction in plaque index scores. Individual study effect sizes ranged from 0.80 (95% CI: −0.73 to 2.33) in Pereira et al. [38] to 4.28 (95% CI: 1.17 to 7.40) in Bapat et al. [28], with weights distributed from 11.4% to 37.9% (refer to Figure 2 for the forest plot visualization, which illustrates study-specific SMDs, confidence intervals, and the pooled estimate).

Heterogeneity was low (I^2^ = 13.7% [95% CI: 0.0% to 82.1%]; τ^2^ = 0.22 [95% CI: 0.00 to 13.66]; H = 1.08 [95% CI: 1.00 to 2.36]), supported by a non-significant Cochran’s Q test (Q = 4.64, df = 4, *p* = 0.327). This minimal variability enhances confidence in the pooled result, revealing consistent efficacy across the included studies.

Clinically, the large pooled effect size (SMD > 0.8) underscores the substantial antimicrobial potential of propolis mouthwash in disrupting oral biofilms, thereby reducing plaque accumulation—a key risk factor for dental caries and early periodontal disease. This intervention may serve as an effective adjunct to mechanical oral hygiene practices, particularly in patients with moderate baseline plaque levels, potentially lowering the incidence of gingival inflammation and associated complications while offering a natural, alcohol-free alternative to conventional rinses.

#### 3.6.3. Publication Bayes

**Figure 3:**
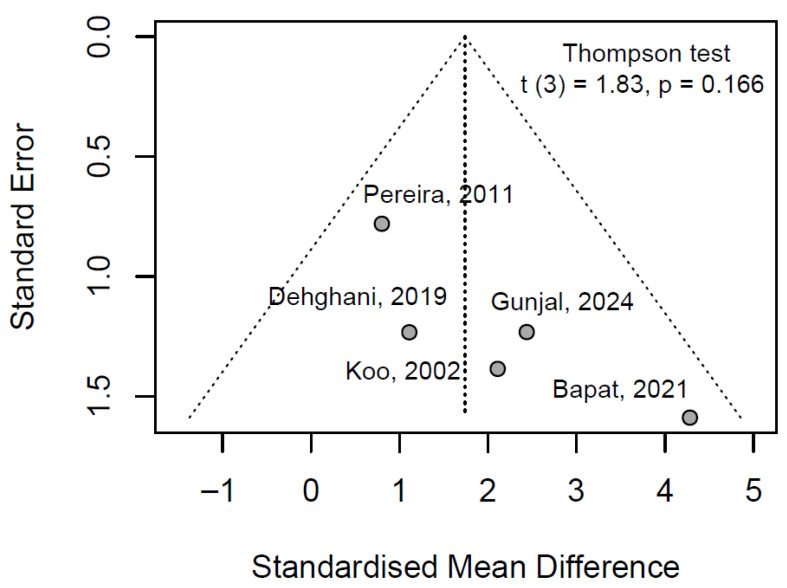
Funnel plot analysis of publication bias in plaque index studies with Thompson–Sharp linear regression test [28,29,31,34,38].

The Thompson test yielded a t = 1.83 (df = 3, *p* = 0.166), with a bias estimate of 3.19 (SE = 1.75). The non-significant *p*-value (*p* > 0.05) indicates no evidence of statistically significant funnel plot asymmetry, indicating that publication bias is unlikely to have substantially influenced the pooled effect estimate. Visual inspection of the funnel plot (Figure 3) supports this finding, displaying a relatively symmetric distribution of study effect sizes around the pooled SMD, with no apparent clustering of smaller studies toward larger effects. This symmetry aligns with the absence of small-study effects or selective reporting, reinforcing the robustness of the meta-analytic results. Clinically, these observations enhance confidence in the inferred efficacy of 5.0% propolis alcohol-free mouthwash for plaque control, as the conclusions appear free from bias-related distortions that could overestimate therapeutic benefits in preventive oral care.

### 3.7. Gingival Index Meta-Analysis

#### 3.7.1. Influence Diagnostics Analysis

Influence diagnostics (Figure S3 in Supplementary Materials) identified the Khabazian et al. [32] study as exerting undue influence on the gingival index meta-analysis, with a studentized residual of −1.65 (approaching the threshold of ±2.0 for outlier detection), DFFITS of −1.04 (indicating substantial shift in the pooled estimate upon omission), and Cook’s distance of 1.04 (>1.0, signifying disproportionate impact on the summary effect). The covariance ratio of 1.37 indicates modest variance adjustment, but collectively, these metrics highlight the study’s outlier status and potential to distort overall results.

Leave-one-out sensitivity analysis sorted by I^2^ (Figure S4 in Supplementary Materials) further demonstrated that excluding Khabazian et al. [32] eliminated heterogeneity entirely (I^2^ = 0.0%), with the pooled SMD increasing to 2.19 (95% CI: 1.10 to 3.29), compared to higher I^2^ values (up to 19.3%) when omitting other studies. The Khabazian et al. [32] was also characterized by the largest influence on the effect size (0.78), underscoring its role in driving inconsistency.

Clinically, the study’s baseline gingival index mean (2.25, SD 0.37) and small sample size (*n* = 10) may reflect a more severe initial inflammation profile or limited statistical power, potentially inflating variability in assessing propolis mouthwash’s anti-inflammatory effects on gingival tissues. Exclusion is thus justified to ensure methodological homogeneity and derive more precise estimates of treatment efficacy in reducing gingival inflammation, thereby supporting reliable clinical recommendations for periodontal management.

#### 3.7.2. Pooled Effect and Between-Study Heterogeneity

**Figure 4:**
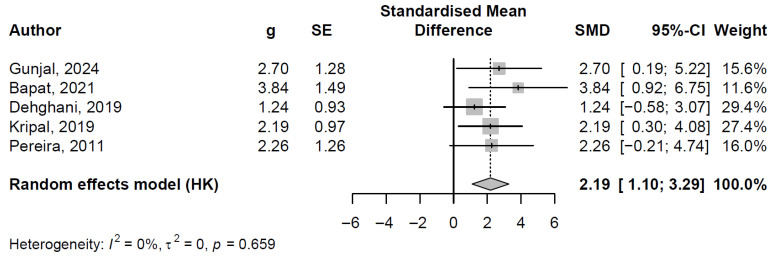
Forest plot of standardized mean differences in gingival index reduction across studies (random effects model) [28,29,31,35,38].

Five randomized controlled trials, involving 129 participants, were synthesized in the meta-analysis of gingival index changes after use of propolis alcohol-free mouthwash, following exclusion of the influential Khabazian et al. [32] study. The random-effects model, utilizing Hedges’ g for standardized mean difference (SMD) with Paule–Mandel tau^2^ estimation and Hartung–Knapp adjustment, produced a pooled SMD of 2.19 (95% CI: 1.10 to 3.29; t = 5.57, *p* = 0.005), denoting a statistically significant decrease in gingival index scores. Study-specific effect sizes varied from 1.24 (95% CI: −0.58 to 3.07) in Dehghani et al. [29] to 3.84 (95% CI: 0.92 to 6.75) in Bapat et al. [28], with random-effects weights ranging from 11.6% to 29.4% (see Figure 4 for the forest plot, depicting individual SMDs, confidence intervals, and the aggregated estimate).

Heterogeneity was absent (I^2^ = 0.0% [95% CI: 0.0% to 79.2%]; τ^2^ = 0 [95% CI: 0.00 to 5.73]; H = 1.00 [95% CI: 1.00 to 2.19]), corroborated by a non-significant Cochran’s Q test (Q = 2.42, df = 4, *p* = 0.659). This lack of variability bolsters the reliability of the pooled findings, implying uniformity in treatment response across the studies.

Clinically, the very large pooled effect size (SMD > 2.0) highlights the potent anti-inflammatory properties of propolis mouthwash in alleviating gingival inflammation. This could translate to reduced bleeding on probing and enhanced periodontal stability, positioning the mouthwash as a valuable adjunctive therapy for patients with mild-to-moderate gingivitis, potentially decreasing progression to periodontitis and improving overall oral health outcomes in routine clinical settings.

#### 3.7.3. Publication Bayes

The test yielded a t-statistic of 1.36 (df = 3, *p* = 0.268), with a bias estimate of 3.44 (SE = 2.54). The non-significant *p*-value (*p* > 0.05) indicates no evidence of statistically significant funnel plot asymmetry, suggesting that publication bias is unlikely to have substantially influenced the pooled effect estimate.

**Figure 5:**
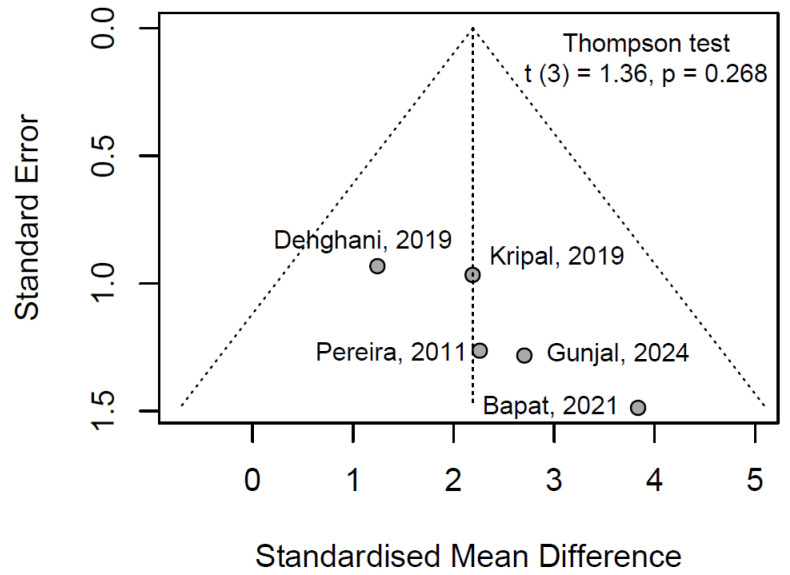
Funnel plot analysis of publication bias in gingival index studies with Thompson–Sharp linear regression test [28,29,31,35,38].

Visual inspection of the funnel plot (Figure 5) supports this finding, displaying a relatively symmetric distribution of study effect sizes around the pooled SMD, with no apparent clustering of smaller studies toward larger effects. This symmetry aligns with the absence of small-study effects or selective reporting, reinforcing the robustness of the meta-analytic results. Clinically, these observations enhance confidence in the inferred efficacy of propolis alcohol-free mouthwash for gingival health, as the conclusions appear free from bias-related distortions that could overestimate therapeutic benefits in preventive oral care.

## 4. Discussion

The use of propolis in periodontal treatments, both as a mouthwash and toothpaste, shows considerable promise in improving key clinical parameters related to gingival and periodontal health.

### 4.1. Mouthwash

Propolis mouthwash has consistently demonstrated effectiveness in reducing both gingival index (GI) and plaque index (PI) across the included studies. Our meta-analysis revealed a pooled standardized mean difference (SMD) of 1.74 for PI and 2.19 for GI, both indicating large effect sizes with minimal heterogeneity (I^2^ = 13.7% for PI and I^2^ = 0.0% for GI). These results support the robust antimicrobial and anti-inflammatory potential of propolis in oral care.

Propolis mouthwash has consistently shown effectiveness in reducing GI and PI across studies, often performing comparably to the gold standard CHX. For example, studies by Bapat et al. [28] and Dehghani et al. [29] found that propolis mouthwash was as effective as CHX in reducing plaque and gingival inflammation. Additionally, propolis mouthwash was observed to improve periodontal indices significantly, such as the Community Periodontal Index (CPI) and papillary bleeding index (PBI), highlighting its anti-inflammatory properties. One notable advantage of propolis over CHX is the lower incidence of side effects, such as tooth staining and altered taste perception, which are commonly associated with prolonged CHX use [47,48]. The COVID-19 pandemic also shifted public interest toward at-home and preventive oral hygiene strategies, including greater openness to natural therapeutic alternatives [49].

Propolis natural composition, rich in flavonoids and phenolic compounds, contributes to its antimicrobial and anti-inflammatory effects. Studies by Kiani et al. [33] and Dodwad et al. [20] illustrate that propolis mouthwash can significantly reduce gingival inflammation and bleeding, even in populations with pre-existing gingivitis. Furthermore, the study by Ercan et al. [30] highlighted the superior efficacy of propolis mouthwash compared to propolis chewing gum, emphasizing the importance of delivery method in achieving optimal clinical outcomes. Additionally, recent research has suggested that propolis may enhance wound healing and cellular regeneration, which can further contribute to periodontal tissue recovery [50].

Despite the positive outcomes, it is important to recognize the variability in study designs, participant populations, and propolis concentrations across all studies included in the systematic review. While the overall trend indicates significant benefits, further standardized research is needed to establish optimal concentrations and long-term effects of propolis mouthwash. Additionally, patient preferences and potential allergies to bee products must be considered when recommending propolis-based treatments.

### 4.2. Toothpaste

Toothpaste containing propolis showed significant benefits in reducing plaque and gingival inflammation, as demonstrated in studies by Bhat et al. [41] and Biria et al. [42]. Propolis toothpaste consistently resulted in lower Modified Gingival and Plaque Index (MGMPI) scores and greater reductions in PI compared to conventional toothpaste. These findings underscore the potential of propolis toothpaste as a superior alternative for oral hygiene maintenance and gingivitis management.

The anti-inflammatory and antimicrobial properties of propolis are likely responsible for its effectiveness in toothpaste formulations. Propolis toothpaste not only reduces plaque accumulation but also enhances gingival health by reducing bleeding indices, as noted in studies like those by Suriamah et al. [46]. The significant decrease in papillary bleeding index observed in these studies supports the role of propolis in promoting healthier gingival tissues.

Moreover, patient compliance and preference for propolis toothpaste need to be assessed to determine its practical application in daily oral care routines. Despite a generally favorable safety profile, propolis is a bee-derived product and can trigger allergic reactions in susceptible individuals. Reported symptoms include local swelling, redness, itching, and, in rare cases, systemic reactions. Clinicians should take a thorough patient history regarding allergies to honey, bee pollen, or bee venom before recommending propolis-containing products. In allergic individuals, alternative interventions should be considered. Regarding future research, it is essential to prioritize larger, well-designed studies to reduce heterogeneity and improve the accuracy of effect size estimates. Future studies should focus on defining optimal concentrations and evaluating patient adherence to propolis-based toothpastes.

### 4.3. Formulation Variability and Clinical Implications

An important factor influencing interpretation of these results is the substantial variability in propolis formulations used in the included studies. Concentrations ranged, extraction methods varied (ethanolic vs. alcohol-free), and products differed in delivery form (mouthwash, toothpaste, chewing gum). These differences may affect potency, bioavailability of active compounds, and patient tolerance. In clinical practice, the lack of standardization makes it challenging to directly apply pooled results. Establishing guidelines for optimal concentration, solvent type, and delivery form would improve reproducibility and facilitate broader clinical adoption.

### 4.4. Practical Relevance for Clinicians

The findings of this review suggest that propolis-based mouthwashes and toothpastes may serve as effective adjuncts in gingivitis management, particularly for patients who are intolerant to CHX or prefer natural products. However, until standardized formulations are available and long-term efficacy is confirmed, propolis should be recommended with clear patient counseling regarding variability in product composition and the importance of monitoring for adverse reactions.

## 5. Limitations

The results of a meta-analysis can be influenced by subjective decisions made by the researchers, such as the selection of studies, inclusion/exclusion criteria, and handling of heterogeneity. The quality assessment revealed that several RCTs were rated as having “some concerns” or “high risk” in certain RoB2 domains, particularly in outcome measurement and lack of blinding. These limitations could have influenced observed effect sizes, especially in smaller trials where subjective scoring of GI and PI is more prone to observer bias. Therefore, while the pooled effects are statistically significant, they should be interpreted with caution. Future studies should employ robust blinding, standardized assessment tools, and adequate sample sizes to minimize risk of bias. Most included trials evaluated outcomes over short follow-up periods. This limits our ability to assess the durability of plaque and gingival improvements or detect delayed adverse effects. Future RCTs should include follow-up periods of at least 6–12 months to evaluate whether benefits are sustained and to confirm long-term safety. Additionally, the relatively small number of high-quality trials underscores the need for further large-scale, randomized controlled trials to confirm these findings.

## 6. Conclusions

In conclusion, propolis, both in mouthwash and toothpaste formulations, presents anti-infective activity and states a promising natural alternative for improving periodontal health. Its effectiveness in reducing plaque and gingival inflammation, coupled with a favorable side effect profile compared to conventional treatments like CHX, makes it an attractive option for patients seeking natural oral care solutions. Propolis can also effectively decrease gingival inflammation and bleeding, without causing tooth discoloration or staining. Due to its antimicrobial properties, propolis treatment is also an alternative treatment option during supportive periodontal therapy. Future research should focus on standardizing propolis concentrations, long-term efficacy, and consider patient preferences to optimize its integration into routine dental practice.

## Figures and Tables

**Table 1:** Summary of study designs and outcomes.

N	Author, Year	Study Design	Inclusion Criteria	Study Groups	Type of Measurements	Timing of Measurements	Results
Mouthwash							
1	Bapat et al., 2021 [28]	Patients were randomized into four groups: hot ethanolic propolis extract, cold ethanolic propolis extract, chlorhexidine (CHX) and distilled water. Patients rinsed twice a day for three months	Age group: 18–22 years old with overall good health	120 participants divided into four equal groups: hot ethanolic propolis extract, cold ethanolic propolis extract, CHX, and distilled water	GI, PI micro-biological analysis	At the beginning of the study and after 15 days, 1 month, and 3 months	Similar plaque reduction was observed in the groups using CHX (0.45), cold ethanolic propolis (0.46) and hot ethanolic propolis (0.47). Propolis was found to be as effective as CHX in reducing GI and PI.
2	Dehghani et al., 2019 [29]	Patients were randomly assigned to two groups: propolis or CHX mouthwash. Patients were asked to rinse their mouths with 15 mL of the liquid for 1 min twice a day after brushing their teeth	Age group: 15–35 years old, with good general health; group: with fixed orthodontic appliances, mild to moderate gingivitis, and completion of a patient satisfaction questionnaire	18 patients in the propolis group and 19 in the CHX group	GI, PI, Community Periodontal Index (CPI)	At the beginning of the study and 22 days apart	The difference between PI (*p* < 0.001), GI (*p* = 0.006), and CPI (*p* = 0.005) before and after propolis administration was statistically significant. However, it was not statistically significant between the two groups of mouthwashes.
3	Dodwad et al., 2011 [20]	Patients randomly assigned to three groups: mouthwash containing propolis, CHX 0.2% or control (saline)	Age group: 18–50 years old with chronic gingivitis, systemically healthy patient	10 patients in the propolis group, 10 patients in the CHX group, and 10 patients in the saline group	GI, PI	At the beginning of the study and at an interval of five days	CHX mouthwash was found to provide better results than propolis and saline in inhibiting plaque formation. Propolis was found to be slightly better than CHX in improving the GI status.
4	Ercan et al., 2015 [30]	Two study groups: a group using propolis mouthwash or a group using chewing gum. Chewing gum was used after meals three times a day for 20 min. The mouthwash group was asked to rinse their mouths with propolis two times a day for 1 min.	Age group: 18–22-year-old students, free of systemic diseases with good oral hygiene	5 patients in chewing gum group, 5 patients in mouthwash group	GI, PI	At an interval of five days	GI and PI in the propolis mouthwash group were significantly lower than in the propolis chewing gum group (*p* = 0.005).
5	Gunjal et al., 2024 [31]	The study group used a mouthwash containing propolis extract, while the control group used an identical mouthwash without the propolis extract and Chlorhexidine mouthwash.	Age group: 8–30 years old patients with Gingival Index > 1, pocket depth ≤ 3 mm, no clinical attachment loss Exclusion criteria: Severe periodontitis	45 adults: three groups of 15 patients: propolis mouthwash, Chlorhexidine mouthwash, and placebo mouthwash—each used by all participants in different phases	GI, PI	Baseline and after 21 day use of each mouthwash, 15 day washout between phases	After 21 days: significant reduction in GI and PI across all groups (*p* < 0.001). Propolis showed greater reduction than Chlorhexidine (*p* < 0.001) and placebo in both GI and PI.
6	Khabazian et al., 2025 [32]	The study group used a chewing gum containing propolis extract, while the comparison group used a mouthwash containing propolis extract.	Age group: patients aged 18–65 with gingivitis, ≥20 teeth, no systemic disease, non-smokers, not pregnant/breastfeeding	20 patients. Two arms study: Propolis chewing gum vs. Propolis mouthwash, assigned randomly (10 patients per group)	GI, PI, Papillary Bleeding Index (PBI)	Baseline and after one week of product use	Both groups had significant reductions in PI (*p* = 0.0001) and GI (*p* = 0.006 for mouthwash).
7	Kiani et al., 2022 [33]	The study group used a mouthwash containing propolis extract. The control group rinsed their mouths with the same mouthwash without propolis extract.	Age group: patients over 18 years old with gingivitis, absence of dental calculus, with a minimum of 20 teeth regardless of wisdom teeth	32 patients: 16 patients in the study group, 16 patients in the control group	PI, PBI, tooth discoloration	At the beginning of the study and after 15 and 30 days	No significant difference between groups in PI (*p* = 0.91). The decrease in papillary bleeding was significantly greater in the propolis group compared to the placebo group (*p* < 0.001).
8	Koo et al., 2002 [34]	Two groups: propolis mouthwash and placebo. During study, the patients withheld from all oral hygiene and rinsed with 20% sucrose solution five times a day to increase dental plaque formation, and with mouthrinse placebo or experimental two times a day.	Age group: patients aged 20–38, good health, at least 24 teeth, no carious lesions, no gingivitis or periodontitis, no crowns or removable orthodontic appliances, no antibiotic therapy in the six months preceding the examination	6 patients, cross-over design	PI	On day four	The PI for the propolis group was significantly lower than in the placebo group.
9	Kripal et al., 2019 [35]	The first group used a 5% propolis mouthwash. The second group used CHX mouthwash, and the control group used saline.	Age group: 18–70 years old patients with chronic gingivitis	45 patients: 15 patients in propolis group, 15 patients in chlorhexidine group, 15 patients in placebo group	GI, PI	At baseline and six weeks after	Significant improvement in clinical parameters (*p* < 0.05).
10	Mallikarjun et al., 2022 [36]	Three groups: propolis mouthwash 20%, CHX 0.2%, and control—saline. The subjects were asked to rinse their mouth for 1 min with 10 mL of the liquid, twice a day for two weeks.	Age group: 18–65 years old, suffering from chronic gingivitis, good general health	20 patients in the propolis group, 20 patients in the CHX group, and 20 patients in the saline group	GI, PI micro-biological examination	At the beginning of the study and on day 15	In all three groups for GI there was no statistically significant difference between groups at the follow-up visits (*p* = 0.204). For PI it was statistically significant (*p* = 0.002).
11	Murray et al., 1997 [37]	Patients were assigned to three groups: a mouthwash containing propolis, a second Periogard^®^ mouthwash with CHX, and placebo mouthwash (without propolis). All patients were instructed to rinse their mouth twice a day with 150 mL of mouthwash for 1 min.	General population, good general health	42 patients: 14 patients in propolis group, 14 patients in CHX group, 14 patients in placebo group	PI	At baseline and five days later	There were significant differences between the mean plaque scores for the active and placebo groups compared to the CHX group (*p* < 0.001). A 14% reduction in plaque was found comparing the test mouthwash with placebo and it was not significant (*p* = 0.19).
12	Pereira et al., 2011 [38]	Patients were instructed to rinse their mouths with 10 mL of alcohol-free mouthwash containing 5.0% Brazilian green propolis for 1 min, immediately after brushing twice a day.	Age group: 18–60 years old, overall good health, at least 20 natural teeth, average GI of at least 1.0, and average PI of at least 1.5	22 patients	GI, PI	During the first visit, after 45 and 90 days	The results showed a significant reduction in GI (*p* < 0.05) and PI (*p* < 0.05) compared to the baseline of the study.
13	Porwal et al., 2018 [39]	Group one received 0.2% CHX, group two received propolis diluted with distilled water (1:1), and group three 3% hydrogen peroxide (1:1). Patients were instructed to rinse their mouths with 10 mL of the liquid twice a day for 15 days.	Age group: 20–40 years old, generally healthy, PI of 4, no clinical attachment loss	10 patients in each group	PI, Modified Gingival Index (MGI)	At the beginning of the study, after 7 and 28 days	All three mouthwashes were effective in reducing GI and PI. CHX 0.2% was most effective in reducing plaque. Propolis was most effective in reducing GI.
**Toothpaste**							
14	Amano et al., 2025 [40]	Patients brushed for one minute with the assigned toothpaste. After a washout period, the procedure was repeated with the alternate toothpaste in a paired crossover design. Participants were randomly assigned to start with either the herbal toothpaste containing Brazilian green propolis (BGP) or the toothpaste without propolis.	Age group: healthy students aged 18–40 years	48 participants (24 males, 24 females), used both toothpastes (with and without BGP) in two separate phases	GI, PI and analysis of oral microbiota	Baseline, after one week, and after two weeks for each toothpaste	BGP toothpaste significantly reduced PI (*p* < 0.05) but not GI.
15	Bhat et al., 2015 [41]	Patients were asked to brush their teeth for 1 min. Baseline plaque levels were recorded. The subjects then withheld oral hygiene for 24 h, and the plaque formation measurements were repeated. After a two-week washout period, the procedure was repeated according to a cross-over design.	Age group: dental students aged 18–22 with at least 24 natural teeth who volunteered and agreed to stop using oral hygiene products for 24 h after their first visit	30 participants	Modified Gingival and Plaque Index (MGMPI)	Baseline and two weeks	Toothpaste with propolis resulted in significantly (*p* < 0.05) lower MGMPI scores than Colgate Total and Miswak toothpastes.
16	Biria et al., 2019 [42]	Patients were randomly divided into two groups: herbal toothpaste with propolis and herbal toothpaste without propolis.	Age group: students aged 24–30 who volunteered to participate in the study and agreed to use the prescribed toothpaste	60 patients: 30 in both groups	PI	At the beginning of the study and after four weeks	There was a significant difference in PI after four weeks (*p* ˂ 0.001).
17	Fereidooni et al., 2014 [43]	Patients were divided into two groups: with propolis toothpaste (Colgate) and regular toothpaste (Colgate).	Age group: students with a mean age of 22 ± 1.2 years	40 participants divided into two groups	PI	At the beginning of the study, at the end of two weeks and after two weeks a third time	The results of this study showed that toothpastes reduce plaque index, and this reduction is more in propolis toothpaste than normal toothpaste.
18	Penmetsa et al., 2023 [44]	Patients were divided into two groups using propolis toothpaste and Colgate toothpaste.	Age group: patients aged 20–35 years, mild to moderate gingivitis, without recent periodontal treatment	40 patients: 20 patients in propolis group, 20 patients in Colgate group	GI, PI	At the baseline and after 30 days	No statistically significant differences were observed between the two groups. Statistically significant differences (*p* ≤ 0.05) occurred in the comparison between baseline and 30 day.
19	Ranjan et al., 2023 [45]	Fifty participants were included in the study. Each participant tested three different toothpastes. Propolis-based toothpaste (Forever Bright), Dabur toothpaste, and Pepsodent.	Age group: healthy dental students aged 24–30, no orthodontic appliances, cavities, probing depth ≤ 3 mm	50 participants (25 male, 25 female); tested three toothpastes: Propolis (Forever Bright), Dabur, and Pepsodent	Modified Gingival Marginal Plaque Index (MGMPI)	At the baseline, after 24 h without brushing, with two-week washout between pastes	After 24 h, Propolis toothpaste showed the lowest plaque accumulation (36.74 ± 2.40). The increase in plaque from baseline to 24h was smallest with Propolis (21.09 ± 1.12), indicating it had the best antiplaque effect.
20	Suriamah et al., 2019 [46]	Two groups: in the test group, patients were instructed to use toothpaste containing propolis, tea tree oil, and sodium monofluorophosphate, while the control group used toothpaste without any natural ingredients.	Age group: students, age group 17–25, good general health, diagnosed gingivitis	40 patients: 20 patients in the study group, 20 in the control group	PI, Papillary Bleeding Index (PBI)	During the first visit and within seven days	Significant decrease (*p* < 0.05) in PI and PBI scores in the test group compared to the control group.

Chlorhexidine (CHX), Community Periodontal Index (CPI), Gingival Index (GI), Modified Gingival Index (MGI), Modified Gingival and Plaque Index (MGMPI), Papillary Bleeding Index (PBI), Plaque Index (PI).

## Data Availability

The original contributions presented in the study are included in the article, further inquiries can be directed to the corresponding author.

## Supplementary Materials

The following supporting information can be downloaded at https://www.mdpi.com/article/10.3390/jfb16090336/s1. Figure S1: Diagnostic plot for plaque index studies highlighting an influential study in red; Figure S2: Leave-one-out analysis of plaque index studies sorted by I2 statistic; Figure S3: Diagnostic plot for gingival index studies highlighting an influential study in red; Figure S4: Leave-one-out analysis of gingival index studies sorted by I2 statistic.
