# Supplementary material for: Propolis as a Natural Remedy in Reducing Dental Plaque and Gingival Inflammation: A Systematic Review and Meta-Analysis
Source: J Funct Biomater. 2025 Sep 8;16(9):336. doi: 10.3390/jfb16090336 (PMC12470411; doi:10.3390/jfb16090336)
Supplement: Supplementary file 1 [file jfb-16-00336-s001.zip › jfb-3813846-supplementary.pdf]

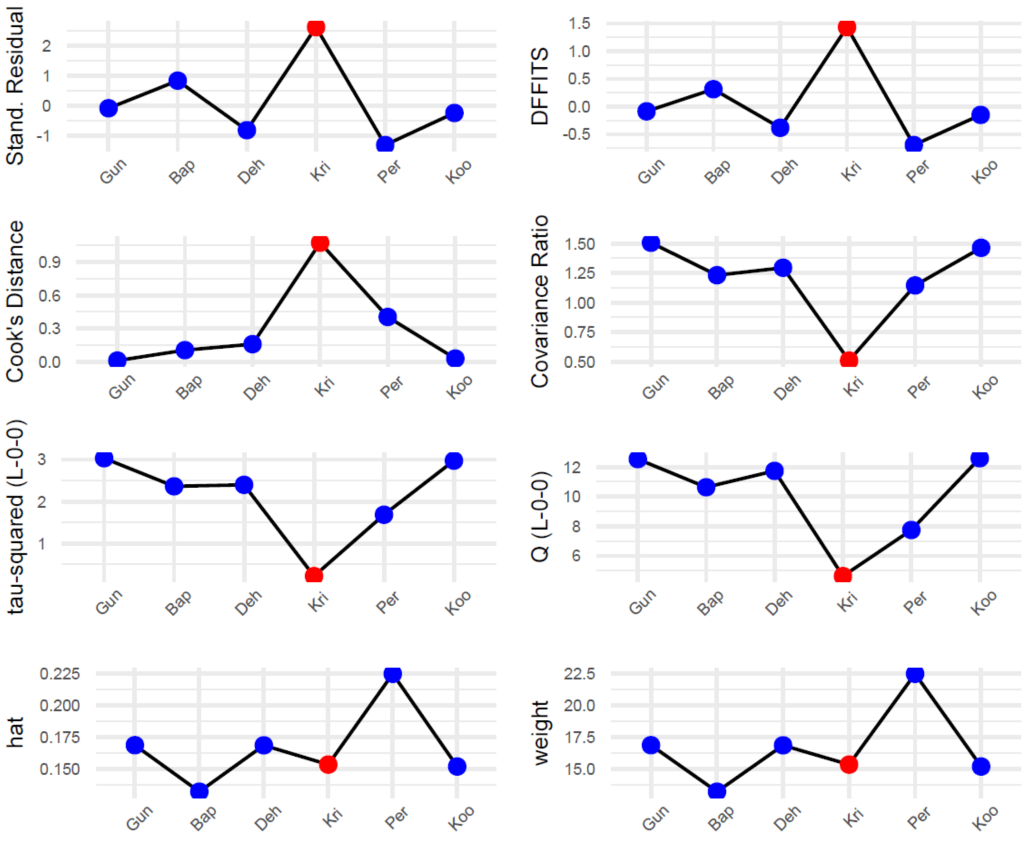

Figure S1 Diagnostic plot for plaque index studies highlighting an influential study in red

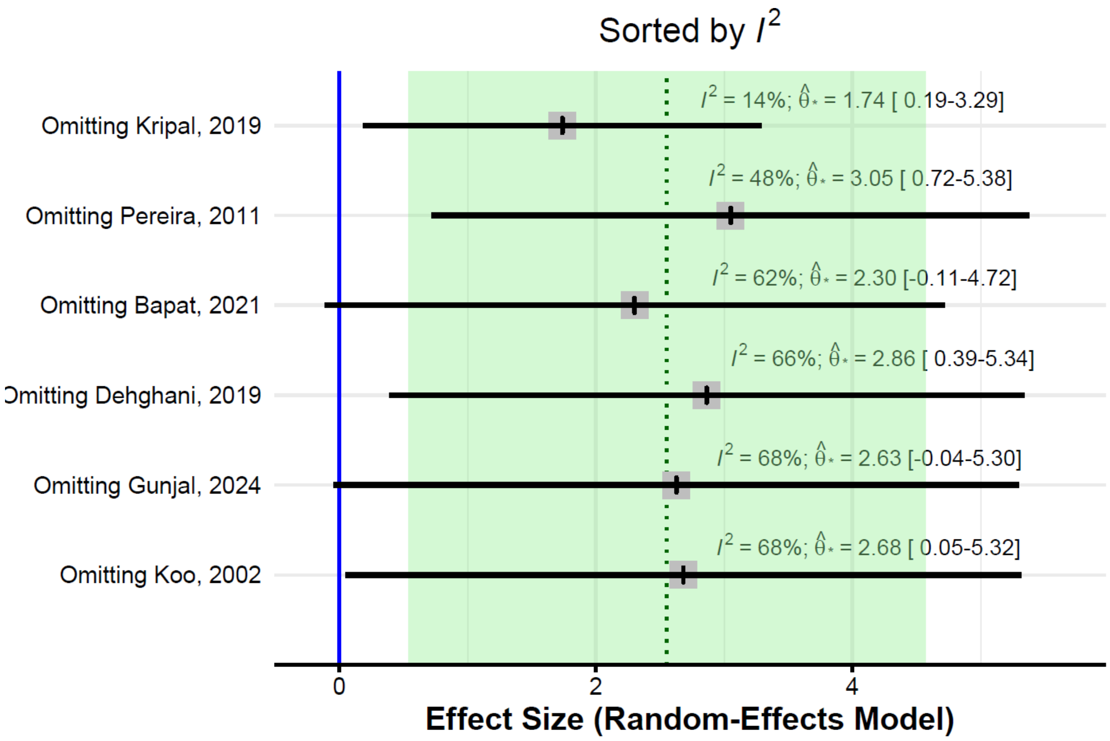

Figure S2 Leave-One-out analysis of plaque index studies sorted by I^2 statistic

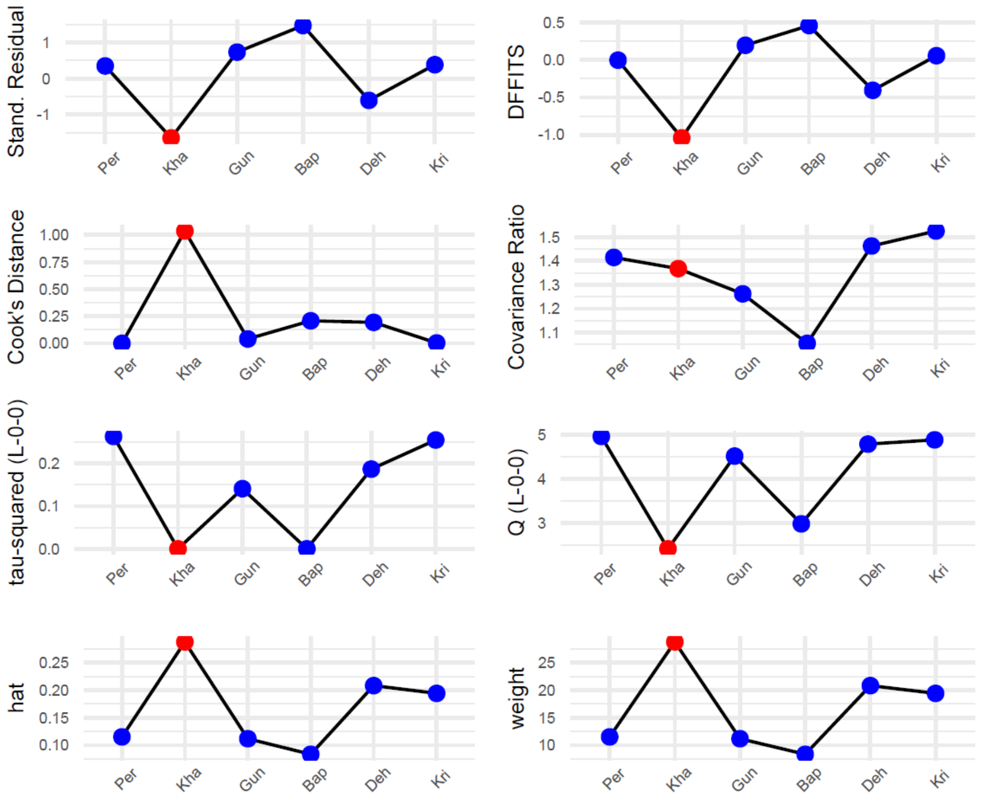

Figure S3 Diagnostic plot for gingival index studies highlighting an influential study in red

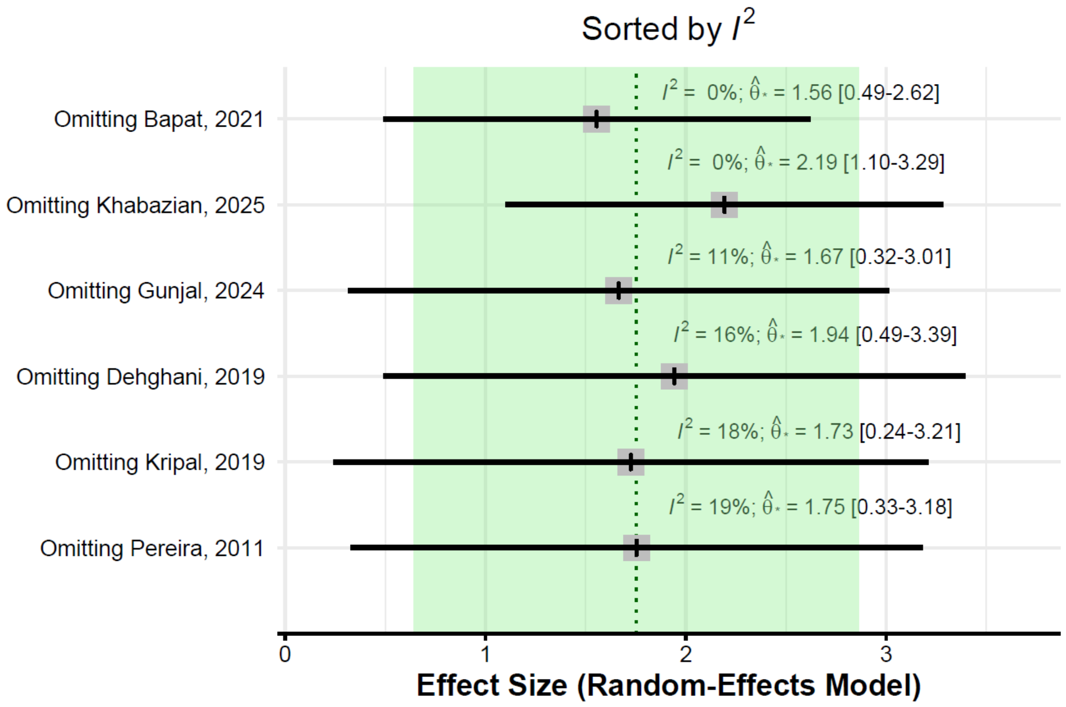

Figure S4 Leave-One-out analysis of gingival index studies sorted by I^2 statistic
